# An Unsuspecting Case of Familial Adenomatous Polyposis (FAP)

**DOI:** 10.7759/cureus.38352

**Published:** 2023-04-30

**Authors:** Akhil Adla, Michael Zheng, Mayank Singhal

**Affiliations:** 1 Medicine, Campbell University School of Osteopathic Medicine, Lillington, USA; 2 Internal Medicine, Cape Fear Valley Medical Center, Fayetteville, USA

**Keywords:** apc gene mutation, gastrointestinal (gi) bleeds, colon cancer prevention, colorectal polyp, familial adenomatous polyposis

## Abstract

Familial adenomatous polyposis (FAP) is an inherited and rare disease that typically manifests in the second decade of life. FAP presents as an asymptomatic disease state in its early stages, which affects the gastrointestinal (GI) tract, making it difficult to diagnose. The disease is characterized by numerous adenomatous polyps in the colon or rectum. Adenomatous polyposis coli (APC) gene mutation allows for the unchecked growth of polyps and provides the path for cancerous proliferation. In FAP, APC mutation inheritance has a moderate preponderance for paternal origin. A family history of FAP is an indicator to start early screening in patients, especially those with a paternal family history of the disease. We present a 21-year-old male patient who presented to the clinic with normal hemoglobin, heme-positive stools, and no family history of FAP or colon cancer. The initial assessment and plan was an endoscopy to look for upper and lower GI causes, but if negative, a further hematologic workup would be required. This case highlighted the need for thorough follow-up and workup of occult GI bleed. Clinical suspicion for FAP should be kept in mind, especially in young patients without a family history and unexplained heme-positive stool. Other findings that could suggest FAP include a personal or family history of extra-colonic manifestations such as fibromas, osteomas, or dentition abnormalities.

## Introduction

Familial adenomatous polyposis (FAP) is a rare and inherited disease impacting the gastrointestinal (GI) tract. The frequency of FAP was reported in one study by the Danish Polyposis Register to be one in 13,528 in the Danish community [1]. Patients typically inherit a defect in the adenomatous polyposis coli (APC) from paternal origin. However, de novo mutations that can occur during gametogenesis or embryogenesis are possible; they only represent a small minority of overall FAP cases. A mutated APC gene results in the formation of thousands of polyps in the colon, which have the potential for malignant transformation. One epidemiology report stated that FAP represents less than 1% of colorectal cancer worldwide [2]. Given 70% of patients classically present with a family history of FAP, the American Gastroenterology Association recommends early surveillance starting at 10 to 12 years for at-risk relatives of patients with FAP [3]. Without early surveillance, the risk of advanced disease and mortality increases. In addition, it is recommended that all first-degree relatives get tested if a patient has confirmed APC mutation.

## Case presentation

A 21-year-old male patient presented to the clinic for evaluation of iron deficiency anemia and heme-positive stools. He reported having worsening iron deficiency since 2016. Several weeks prior, the patient underwent surgical management of a pilonidal cyst. During pre-op, the patient was noted to have iron deficiency anemia with heme-positive stools.

The patient at the time of the visit denied any history of GI complaints or overt GI bleeding. He only noted occasional episodes of what he described as "red gelatinous material" in his stool from 2016 to 2017, which he attributed to his red-colored food intake. The patient’s pertinent past medical history included iron deficiency anemia. His surgical history included pilonidal cyst drainage and odontoma removal. The family history was negative for FAP or colorectal cancer, but the paternal grandfather had a history of prostate and esophageal cancer. Occasional alcohol use was only noted in social history.

On examination, the patient was afebrile and normotensive, with a pulse of 84 bpm and a BMI of 25.0. A physical exam showed a well-developed, well-nourished patient who was in no apparent distress. Skin and eye exams did not show pallor or jaundice. The pulmonary exam was non-labored with normal breath sounds. GI examination revealed normal bowel sounds, no masses or tenderness, negative organomegaly, and absent abdominal distention.

After examination, causes of iron deficiency were discussed with the patient including celiac disease, upper GI causes, gastritis, and peptic ulcer disease. Given the heme-positive stool, an upper endoscopy was recommended initially to rule out upper GI causes. In addition, a colonoscopy following a negative upper endoscopy was discussed with further hematologic workup if both studies were negative.

**Figure 1:**
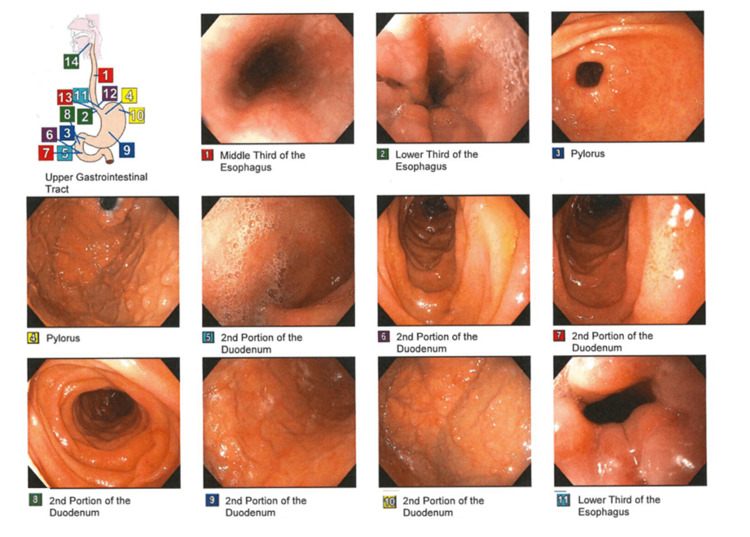
Esophagogastroduodenoscopy (EGD) of the patient Images from the EGD are labeled by number with corresponding anatomical locations. Polyps were found in the duodenum and upper gastrointestinal tract.

**Figure 2:**
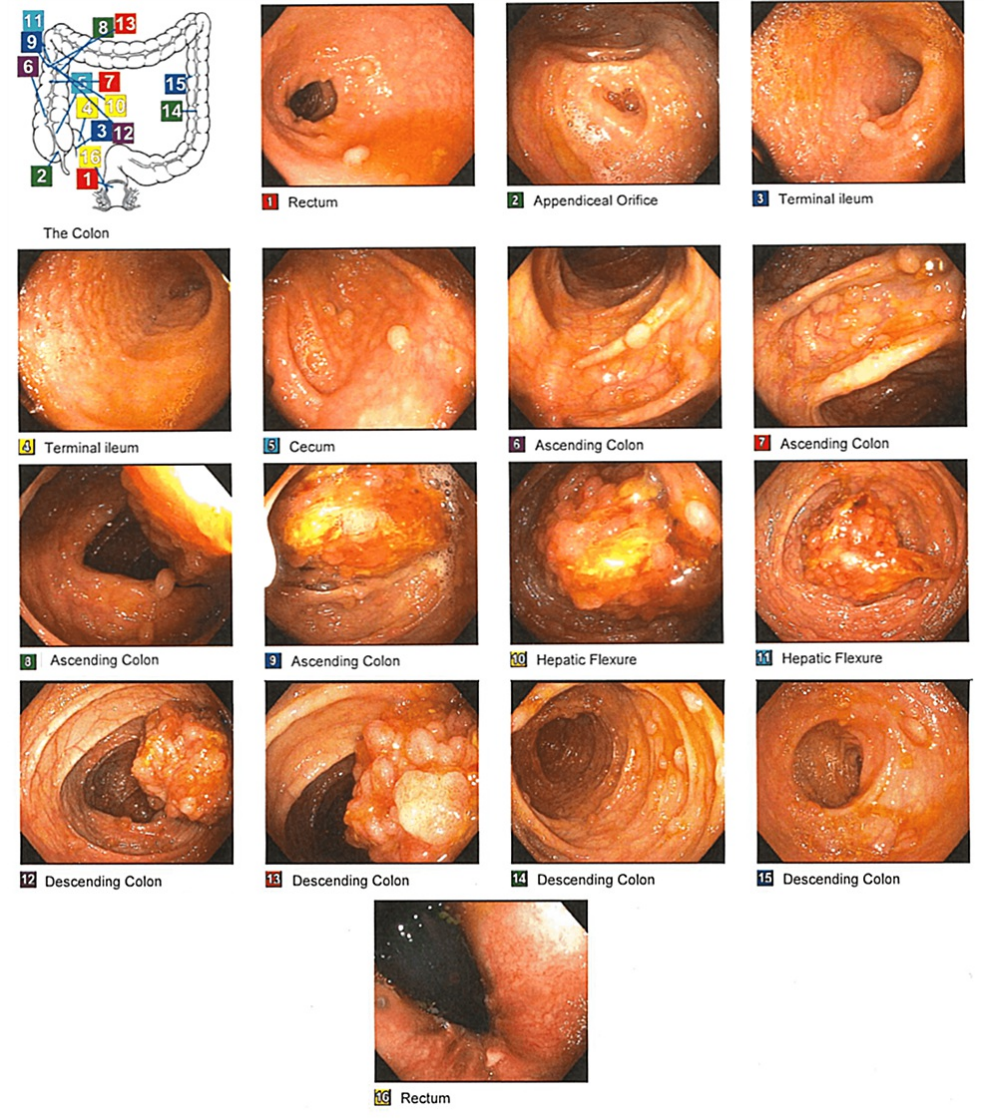
Colonoscopy of the patient Images from the colonoscopy are labeled by number with the corresponding anatomical locations. Hundreds of polyps were revealed that were unable to be removed.

Esophagogastroduodenoscopy (EGD) showed gastritis but was negative for *Helicobacter pylori*, ulcers, or other lesions for the cause of bleeding (Figure 1). However, multiple fundic polyps measuring <1-2 mm were noted and removed. Pathology of the removed polyps showed low-grade dysplasia. EGD was followed by a colonoscopy that showed multiple sessile and semi-sessile polyps numbering greater than 100 in the entire colon (Figure 2). An isolated large polyp, measuring 3-4 cm, was found in the ascending colon. The polyp was noted to be multilobulated, and biopsies were taken with cold forceps for histology. Pathology later confirmed the removed polyps as tubular adenomas. The patient was notified of the findings and was referred to both a polyposis clinic and a genetic clinic at a major academic center. Genetic testing revealed a pathogenic variant of the APC gene confirming FAP diagnosis. All first-degree relatives were recommended to undergo testing for FAP. At the academic center, a two-stage colectomy with an ileoanal pouch was recommended.

## Discussion

FAP is an uncommon diagnosis, especially without a family history. About 30% of patients with FAP are the first person in the family to present with the condition [4]. Given the patient's lack of symptoms and history, it was not high on the differential. However, about 25%-30% of FAP patients have "de novo" mutations without any family history [1]. Differential diagnosis on presentation included celiac disease, iron deficiency, gastritis, and upper GI ulcers. FAP patients present with blood in stool and general symptoms. FAP increases the risk for colorectal cancer, thyroid cancer, and desmoid tumors [4]. The main cause of mortality in FAP is colorectal cancer. If left untreated, the likelihood of developing colorectal cancer is nearly 100%. The average age of diagnosis for colorectal cancer in FAP patients is 39 years old. Desmoid tumors (DT) occur in about 10%-20% of FAP patients and are the second leading cause of death. DTs are benign tumors with aggressive behavior of invading adjacent tissues [5]. The risk of thyroid cancer is 1%-2% with FAP, specifically papillary thyroid cancer.

Patients with a risk of FAP should undergo annual colonoscopy screening at 10-12 years old. Lifelong screening is recommended for those carrying the APC mutation. In our patient, findings of greater than 100 polyps indicate a diagnosis of FAP with further workup including genetic screening. Additional screening includes upper endoscopy and thyroid ultrasound for extra-colonic malignancies. First-degree relatives should be screened as was the case in Dalavi et al., where the patient's two children were screened by colonoscopy and found to have multiple polyps [3].

The primary goal of management beyond supportive treatments to maintain a good quality of life is cancer prevention. Management of FAP involves surgical intervention with the removal of all or portions of the colon. Even with a total colectomy, there is a possibility of cancer recurrence in the ileal pouch or rectal cuff. In one study, rectal cancer was found in 16.6% of patients that underwent total colectomy with ileal-rectum anastomosis [6]. Routine screening is essential in monitoring disease progression and cancer prevention.

## Conclusions

The initial assessment did not include FAP as a potential cause due to the patient’s lack of family history and age. The case highlighted the importance of using colonoscopy sooner in patients with unexplained causes of heme-positive stools. Some indications for colonoscopy by the American Society for Gastrointestinal Endoscopy (ASGE) include unexplained lower GI bleeding, unexplained iron deficiency anemia, family history of colorectal cancer, or a history of inflammatory bowel disease.
